# Impact on Patients’ Treatment Outcomes of XpertMTB/RIF Implementation for the Diagnosis of Tuberculosis: Follow-Up of a Stepped-Wedge Randomized Clinical Trial

**DOI:** 10.1371/journal.pone.0123252

**Published:** 2015-04-27

**Authors:** Anete Trajman, Betina Durovni, Valeria Saraceni, Alexandre Menezes, Marcelo Cordeiro-Santos, Frank Cobelens, Susan Van den Hof

**Affiliations:** 1 Federal University of Rio de Janeiro, Rio de Janeiro, Brazil; 2 McGill University, Montreal, Canada; 3 Rio de Janeiro City Health Secretariat, Rio de Janeiro, Brazil; 4 Dr. Heitor Dourado Vieira Foundation of Tropical Medicine, Manaus, Brazil; 5 Global Health Strategies, Rio de Janeiro, Brazil; 6 Amazonas State University, Manaus, Brazil; 7 Amsterdam Institute for Global Health and Development, Academic Medical Center, Amsterdam, Netherlands; 8 KNCV Foundation, The Hague, Netherlands; The Foundation for Medical Research, INDIA

## Abstract

**Introduction:**

The impact on treatment outcomes of XpertMTB/RIF, a molecular-based test that provides rapid diagnosis of tuberculosis (TB) and rifampicin resistance with high accuracy, has not been reported despite its adoption in a few countries. We here report treatment outcomes in a step-wedged cluster randomized trial for patients diagnosed with XpertMTB/RIF compared to patients diagnosed with sputum smear examination in public health facilities in Brazil.

**Methods:**

Treatment outcome data were added to the trial database of patients diagnosed from 4 February to 4 October 2012, and crosschecked with data from the national mortality and the drug-resistant TB registers. Treatment outcomes in the intervention (n=2232) and baseline (n=1856) arms were compared using a multilevel regression model.

**Results:**

Unfavourable outcomes were frequent in both arms, mainly due to loss to follow-up (16%). Overall unfavourable outcomes were not reduced in the intervention arm (29.6% versus 31.7%, OR=0.93; 95%CI=0.79-1.08). However, the overall TB-attributed death rate was lower in the intervention arm (2.3% vs. 3.8%). Adjusted for HIV status, age group and city, the intervention resulted in a 35% decrease in TB-attributed deaths (OR=0.65, 95%CI=0.44-0.97).

**Conclusions:**

The proportion of patients successfully treated did not increase with Xpert MTB/RIF implementation, with high loss to follow-up rates in both arms. We did observe a 35% reduction in TB-related mortality, which we hypothesize may be explained by less advanced disease among the smear-negative patients diagnosed by Xpert. In conclusion, XpertMTB/RIF introduction did not improve TB treatment outcomes in Brazil.

**Trial Registration:**

clinicaltrials.gov NCT01363765

## Introduction

Novel tests for the diagnosis of tuberculosis (TB) and drug resistance (DR) have been developed in the last decade. The XpertMTB/RIF (Xpert, Cepheid, Sunnyvale, CA, USA) is a real-time polymerase chain reaction-based test performed within two hours on an semi-automated platform.[1] With this test, clinicians can quickly be informed about TB diagnosis and resistance to rifampicin, a good marker of multidrug-resistant (MDR) TB.[2] Because of the rapid and accurate detection of TB and of rifampicin resistance,[2] Xpert has the potential to reduce TB morbidity, mortality and transmission.[3]

Since the World Health Organization endorsement for its use,[4] a number of diagnostic trials have been conducted in high TB-burden countries, two of which we know the results.[5,6] These studies have demonstrated increased rates of TB confirmation with Xpert but no impact on overall TB notifications, including in the setting where this accurate and rapid test became available in routine practice.[6] One of the possible explanations is a tendency among physicians in high-burden countries to treat TB empirically (i.e., based on clinical and radiological criteria).[7] More importantly, the one trial that assessed patients’ morbidity and mortality reported no such impact of Xpert implementation.[6,8]

We aimed to evaluate the effect of Xpert implementation on patients’ treatment outcomes in public health primary care clinics of two cities with a high TB incidence rate (over 90/100,000 inhabitants) in Brazil. We hypothesized that a more sensitive and rapid tool for confirmation of TB diagnosis could (i) reduce treatment loss to follow up, assuming that patients would more easily commit to a 6-month treatment regimen if their disease was laboratory confirmed, (ii) increase successful treatment outcome rates, assuming that less false positive clinical diagnosis would result in less inadequate empiric treatment and (iii) reduce other unfavourable outcomes by early detection of rifampicin resistant TB.

## Methods

### Ethical statement

The study was approved by the National Ethics Board (Comissão Nacional de Ética em Pesquisa-CONEP, #494/2011), the Rio de Janeiro Municipal Health Department Review Board (CEP SMS # 236/11) and the Tropical Medicine Foundation of Manaus Review Board (CEP FMT/HVD, November 24, 2011). The need for informed consent was waived by the three ethical boards based on the researchers’ claim that this was an implementation of a diagnostic test in routine practice and only routine reporting data were used for the analysis.

We previously reported on the data collection methods for a stepped-wedge trial[9] comparing TB diagnosis and notification rates using a single sputum sample Xpert assay (intervention arm) versus two sample sputum smear microscopy (baseline arm).[5] In brief, data from patients notified with TB in the Brazilian national TB information system (SINAN) were linked to test results in the laboratory information system (GAL). Automated[10] and manual linkage was performed using name of the patient, name of the mother, sex and date of birth. The study was conducted from February 4 to October 4 2012 in Rio de Janeiro and Manaus, two cities with high TB incidence rates (over 90/100,000 inhabitants in 2012). Xpert MTB/RIF was implemented in 14 laboratories that diagnose 70% of patients in both cities. Each laboratory contributed to both arms of the trial (smear examination or Xpert MTB/RIF as the primary laboratory test), but the time point at which a lab moved from the smear arm to the Xpert arm was randomized. Because of expectedly low positive predictive value for a Xpert rifampicin resistance result, no immediate changes in management of genotypically detected rifampicin-resistant cases were recommended during the study period: patients were started on first-line drugs (FLD) while waiting for culture and phenotypic drug-susceptibility testing (DST) results. Treatment outcomes reported in SINAN were added to the study database in January 2014 (9 months after the last Xpert MTB/RIF test performed within the trial period, corresponding to a minimum follow up period of 15 months and a maximum of 23 months).

For the present study, we also linked data from cases in SINAN to the mortality information system (SIM) and to the resistant-TB system (SITE-TB), using the same linkage strategy as used previously with the laboratory information system GAL. The final anonymized database is available on request.

Treatment outcomes[11] registered in the TB information system SINAN are: (1) successful; (2) loss to follow-up; (3) TB-attributed death; (4) death from other causes; (5) change of diagnosis; (6) transfer out and (7) suspicion of drug resistance. Successful treatment includes cure and treatment completion without evidence of failure. The National TB Program defines loss to follow-up when the patient has missed at least 30 days of treatment or when no contact is achieved despite calls, visits and telegrams for at least 60 days. For all deaths, cause of death (TB-related or not) was double-checked with the mortality information system (SIM). TB-attributed death was considered whenever TB appeared as the primary or a secondary cause of death in the death certificate. Discrepancies between mortality and notification databases were resolved by using information from the death certificate. Failure is not an outcome option anymore in the Brazilian notification system. Patients who fail FLD treatment are referred to more specialized centres (transferred out) because of suspected drug resistance, as are those with intolerance to TB drugs. When we found transferred-out patients in the referral resistance register (SITE-TB), we re-categorized them as “suspicion of drug resistance”. If not found in SITE-TB the outcome “transferred out” was maintained. Referral for drug resistance suspicion may occur either during treatment or after failure of FLD treatment. For those with confirmed resistance, final treatment outcomes were unknown by the time of this analysis due to the 18–24 month treatment regimen.

The treatment outcomes death from any cause, default, transfer out and suspicion of drug resistance were classified as unfavourable outcomes.

Time to death and to the diagnosis of resistance was estimated as days elapsed since the date of notification to the outcome registered (date of death according to the death certificate or date of SITE-TB registration).

We compared treatment outcomes for patients diagnosed in the intervention arm (diagnosis with Xpert) with those for patients treated in the baseline arm (diagnosis with smear microscopy). To assess the effect of differences in test accuracy for smear microscopy and Xpert MTB/RIF, we stratified our analyses by type of diagnosis: (1) confirmed TB was considered when at least one sputum smear was positive in the baseline arm or when the Xpert assay was positive in the intervention arm, (2) empirically diagnosed TB with a negative test result, when tests were performed but smear or Xpert results were negative; and (3) empirically diagnosed with no test results, when no test results were found in GAL.

Proportions were compared using the chi-square test and the Wilcoxon test was used to compare medians and their dispersion. The magnitude of the effect of the intervention was measured by the odds ratio (OR) and its 95% confidence interval (95%CI), adjusted for sex, age group, city, HIV status and diagnosis status (positive test result, negative test result, no test result) in a multilevel logistic regression model, accounting for within-cluster correlation, using robust variance estimators. Laboratories drain patients from a few clinics located in their neighbourhood, thus laboratories represent city area (especially in Rio de Janeiro; in Manaus one of the clinics receives patients from all over the city). The main determinant of interest—study arm- and all potential confounders (sex, age, city, HIV, type of diagnosis), were included in the multivariable model. Where possible, the model was refined by backward elimination, guided by the fit of the model and confounding effects. Interactions between HIV and diagnosis status were explored for with regard to effect of the main determinant, study arm.

Finally, because the true outcome of “transferred out” patients—considered as unfavourable—is unknown, sensitivity analyses were performed including them as favourable outcomes and excluding them from the analyses. In an additional sensitivity analysis, we combined non-tested and negative-tested patients in one category of non-bacteriologically confirmed TB.

Time to treatment outcome was calculated as (i) time between sputum processing date (obtained from GAL) and treatment outcome date (obtained from SINAN) for all notified patients where a sputum sample had been submitted for laboratory testing, and (ii) time between date of notification (as proxy for date of start of treatment) and treatment outcome date, both obtained from SINAN, for all notified patients. Cluster-averaged mean delays were compared with the Wilcoxon signed-rank test.

## Results

**Fig 1:**
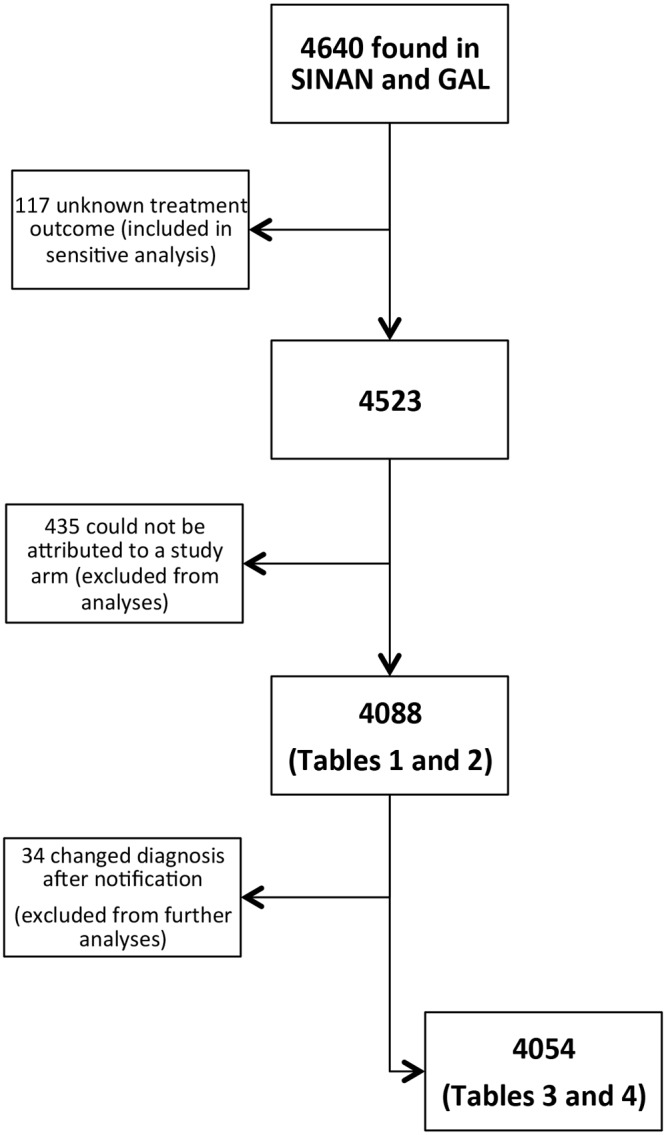
Flowchart of patients included in the present analyses. Flowchart showing study inclusion in baseline (smear examination) and intervention (Xpert MTB/RIF) arm.

Out of 4640 patients notified to SINAN, treatment outcome was registered for 4523 (97.5%), of whom 4088 (90.4%) could be allocated to a study arm (Fig 1). The 435 patients who could not be allocated to a study arm were from Manaus. Their records were not found in GAL, and therefore could not be attributed to a laboratory and study arm. Out of the 4088, 2232 (54.6%) were diagnosed in the intervention arm. Patients’ characteristics by arm are displayed in Table 1. As a result of randomization, the three labs in Manaus started the intervention in the first three months of the study, which explains the large proportion of samples in the intervention arm for Manaus. TB diagnosis had been confirmed by a positive test result for 2963 (72.5%) patients, while 713 (17.4%) patients were empirically treated despite a negative test result, and 412 (10.1%) were empirically treated with no test result. As expected and previously reported,[5] more patients in the intervention arm (76.2% versus 68.0%) had their diagnosis bacteriologically confirmed. Cross linkage between the notification information system (SINAN) and the mortality information system (SIM) confirmed all deaths from TB reported.

**Table 1:** Patients’ characteristics in the baseline and intervention arms.

Characteristics	Baseline	Intervention
	(n = 1856)	(n = 2232)
***Sex***		
Female	666 (35.9%)	778 (34.9%)
Male	1190 (64.1%)	1454 (65.1%)
***Age group (years)***		
0–14	52 (2.8%)	41 (1.8%)
15–29	967 (52.1%)	1194 (53.5%)
30–59	619 (33.4%)	728 (32.6%)
≥60	218 (11.7%)	269 (12.1%)
***HIV status***		
Negative	1017 (54.8%)	1023 (53.9%)
Positive	201 (10.8%)	198 (8.9%)
Unknown	638 (34.4%)	831 (37.2%)
***City***		
Manaus	159 (8.6%)	627 (28.1%)
Rio	1697 (91.4%)	1605 (71.9%)
***Type of diagnosis***		
Confirmed*	1262 (68.0%)	1701 (76.2%)
Clinically diagnosed, negative test†	381 (20.5%)	332 (14.9%)
Clinically diagnosed, no test result	213 (11.5%)	199 (8.9%)

*Statistically significant difference between column proportions at the 0.05 p-value level

*Positive smear in baseline arm and positive Xpert in intervention arm

^†^Negative smear in baseline arm and negative Xpert in intervention arm

Table 2 shows the outcomes by arm, stratified by type of diagnosis. The proportion of patients with a successful treatment outcome was similar in both arms; overall (70.4% vs 68.3%) and in all three groups of type of diagnosis. In contrast, the proportion of patients with TB-attributed death was lower in the intervention arm, both overall (2.3% vs. 3.8%) and in the three groups, and this was confirmed in the multilevel model (see below). The largest proportion of patients with an unsuccessful outcome was due to loss to follow-up, which was not changed by the intervention (16.2% vs. 15.9%).

**Table 2:** Patients’ treatment outcomes in the baseline and intervention arm, stratified by type of diagnosis.

Diagnosis	Outcome	Baseline	Intervention
		(n = 1856)	(n = 2232)
***Overall*** (n = 4088)	Successful	1267 (68.3%)	1571 (70.4%)
	Loss to follow-up	300 (16.2%)	356 (15.9%)
	TB-attributed death	71 (3.8%)	52 (2.3%)
	Other deaths	38 (2.0%)	36 (1.6%)
	Transfer out	145 (7.8%)	160 (7.2%)
	Change of diagnosis	16 (0.9%)	18 (0.8%)
	Resistance	19 (1.0%)	39 (1.7%)
***Confirmed*** (n = 2963)	Successful	860 (68.1%)	1180 (69.4%)
	Loss to follow-up	206 (16.3%)	293 (17.2%)
	TB-attributed death	46 (3.7%)	39 (2.3%)
	Other deaths	14 (1.1%)	19 (1.1%)
	Transfer out	110 (8.7%)	124 (7.3%)
	Change of diagnosis	8 (0.6%)	7 (0.4%)
	Resistance	18 (1.4%)	39 (2.3%)
***Clinically diagnosed (negative tests)*** (n = 713)	Successful	256 (67.2%)	245 (73.8%)
	Loss to follow-up	56 (14.7%)	36 (10.8%)
	TB-attributed death	17 (4.5%)	8 (2.4%)
	Other deaths	20 (5.2%)	13 (3.9%)
	Transfer out	26 (6.8%)	22 (6.6%)
	Change of diagnosis	5 (1.3%)	8 (2.4%)
	Resistance	1 (0.3%)	0 (0.0%)
***Clinically diagnosed (no test result)*** (n = 412)	Successful	151 (70.9%)	146 (73.4%)
	Loss to follow-up	38 (17.8%)	27 (13.6%)
	TB-attributed death	8 (3.8%)	5 (2.5%)
	Other deaths	4 (1.9%)	4 (2.0%)
	Transfer out	9 (4.2%)	14 (7.0%)
	Change of diagnosis	3 (1.4%)	3 (1.5%)
	Resistance	0 (0.0%)	0 (0.0%)

Thirty-four patients whose diagnosis was changed after notification were excluded from further analysis (Fig 1). Thus, the effect of intervention was analysed among 4054 patients. In adjusted analysis, overall unfavourable outcome was not significantly reduced in the intervention arm (aOR = 0.93; 95%CI = 0.79–1.08, Table 3). Young adults (15–29 years) and those clinically diagnosed had a lower risk of unfavourable outcomes. Residence in Rio and a positive HIV status was associated with an increased risk of unfavourable outcomes. A positive HIV status also was associated with an increased risk of TB-related death (aOR = 14.1, 95%CI 9.1–26.5, Table 4). Adjusted for HIV status and age group, the intervention resulted in a 35% decrease in TB-attributed deaths (OR = 0.65, 95%CI = 0.44–0.97). There was no interaction between type of diagnosis and HIV status.

**Table 3:** Characteristics associated with unfavourable outcomes in a multilevel logistic regression model.

Characteristics	Unfavorable†	Successful	crude OR	aOR
	(n = 1216)	(n = 2838)	(95%CI)	(95%CI)
***Sex***				
Female	875 (33.4%)	1752 (66.8%)	1.6 (1.4–1.8)	**1.6 (1.3–1.8)**
Male	341 (23.9%)	1086 (76.1%)	1 (reference)	1 (reference)
***Age group (years)***				
0–14	18 (19.6%)	74 (80.4%)	0.7 (0.4–1.3)	0.7 (0.4–1.2)
15–29	726 (33.8%)	1424 (66.2%)	1.6 (1.3–2.0)	**1.6 (1.2–2.0)**
30–59	355 (26.7%)	977 (73.3%)	1.1 (0.9–1.4)	1.1 (0.8–1.4)
≥60	117 (24.4%)	363 (75.6%)	1 (reference)	1 (reference)
***HIV status***				
Negative	468 (21.2%)	1735 (78.8%)	1 (reference)	1 (reference)
Positive	174 (43.7%)	224 (56.3%)	2.9 (2.4–3.8)	**3.1 (2.4–3.9)**
Unknown	574 (39.5%)	879 (60.5%)	2.6 (2.2–3.0)	**2.6 (2.2–3.0)**
***City***				
Manaus	225 (28.7%)	560 (71.3%)	1 (reference)	1 (reference)
Rio	991 (30.3%)	2278 (69.7%)	1.05 (0.8–1.4)	**1.3 (1.01–1.8)**
***Type of diagnosis***				
Confirmed*	908 (30.8%)	2040 (69.2%)	1 (reference)	1 (reference)
Clinically diagnosed, negative test^§^	199 (28.4%)	501 (71.6%)	0.89 (0.73–1.06)	0.84 (0.69–1.02)
Clinically diagnosed, no test result	109 (26.8%)	297 (73.2%)	0.81 (0.64–1.03)	**0.7 (0.59–0.96)**
***Arm***				
Baseline	573 (31.1%)	1267 (68.9%)	1 (reference)	1 (reference)
Intervention	643 (29.0%)	1571 (70.9%)	0.92 (0.79–1.06)	0.93 (0.79–1.08)

*Positive smear in baseline arm and positive Xpert in intervention arm.

^§^Negative smear in baseline arm and negative Xpert in intervention arm.

^†^Unfavourable = loss to follow up, death from any cause, transfer out and resistance.

**Table 4:** Characteristics associated with TB-attributed death in a multilevel logistic regression model.

Characteristics	TB-attributed death	Successful)	crude OR	aOR
	(n = 123)	(n = 2838)	(95%CI)	(95%CI)
***Sex***				
Male	87 (4.7%)	1752 (95.3%)	1.5 (1.01–2.2)	-
Female	36 (3.2%)	1086 (96.8%)	1.0 (reference)	
***Age group (years)***				
0–14	1 (1.3%)	74 (98.7%)	0.19 (0.03–1.5)	0.13 (0.02–0.98)
15–29	50 (3.4%)	1424 (96.6%)	0.51 (0.31–0.84)	**0.41 (0.24–0.68)**
30–59	47 (4.6%)	977 (95.4%)	0.70 (0.42–1.2)	**0.53 (0.31–0.90)**
≥60	25 (6.4%)	363 (93.6%)	1.0 (reference)	1.0 (reference)
***HIV status***				
Negative	26 (1.5%)	1735 (98.5%)	1.0 (reference)	1.0 (reference)
Positive	44 (16.4%)	224 (83.6%)	14.5 (8.5–24.6)	**14.1 (9.1–26.5)**
Unknown	53 (5.7%)	879 (94.3%)	4.2 (2.6–6.9)	**4.4 (2.7–7.1)**
***City***				
Manaus	22 (3.8%)	560 (96.2%)	1.1 (0.70–1.8)	1.5 (-8.1–0.57)
Rio	101 (4.2%)	2278 (95.8%)	1.0 (reference)	1.0 (reference)
***Type of diagnosis***				
Confirmed*	85 (4.0%)	2040 (96.0%)	1.0 (reference)	-
Clinically diagnosed, negative test^§^	25 (4.8%)	501 (95.2%)	1.2 (0.75–1.9)	
Clinically diagnosed, no test result	13 (4.2%)	297 (95.8%)	1.1 (0.58–1.9)	
***Arm***				
Baseline	71 (5.3%)	1267 (94.7%)	1.0 (reference)	1.0 (reference)
Intervention	52 (3.2%)	1571 (96.8%)	0.59 (0.41–0.85)	**0.65 (0.44–0.97)**

OR = odds ratio, aOR = adjusted odds ratio

*Positive smear in baseline arm and positive Xpert in intervention arm

^§^ Negative smear in baseline arm and negative Xpert in intervention arm

We did two sensitivity analyses (data not shown). First, when analyses were repeated combining the two groups of non-bacteriologically confirmed patients (those with negative test result and no test result) to those with a confirmed diagnosis, the results were very similar. Second, considering transferred-out as a favourable outcome or excluding transferred-out patients from the analyses did not change the findings.

Time from laboratory testing to death from TB and time from treatment initiation to death from TB was similar in both arms. Median time from treatment initiation to TB-death was 93.5 days, interquartile range (IQR) = 65.9–150.5 days in the intervention arm vs. 103 (IQR = 63–123) days in the baseline arm, p = 0.42).

Time from treatment initiation to referral and registration in the drug resistance system (SITE-TB) was not statistically different in both arms [142.5 (IQR = 123–161) days in the intervention versus 163.8 (IQR = 116–242.8) days in the baseline arm, p = 0.12]. However, time from test result in GAL to SITE-TB notification was significantly shorter in the intervention arm [99.6 (IQR = 70.7–153.3) days vs. 166.3 (IQR = 91.3–268.2) days, p = 0.046)]. Among laboratory confirmed TB patients with rifampicin-resistant TB, 5 (12.8%) out of 39 in the intervention arm were registered in the resistance information system only after the end of FLD treatment vs. 9 (50%) out of 18 in the baseline arm (p = 0.02). The rest were registered during TB treatment (i.e. within six months after initiation of treatment).

## Discussion

In the present pragmatic trial of Xpert implementation in two large cities in Brazil, we observed, among those started on treatment, an overall reduction in TB-attributed deaths and a reduction in referral delays for potential drug-resistant TB patients. However, treatment success rates were similar when comparing those diagnosed using an algorithm with smear microscopy versus one with the more sensitive Xpert technology. So although the proportion of bacteriologically confirmed cases was higher in the intervention arm, the distribution of treatment outcomes only differed with regard to TB-attributed deaths.

Despite the reduction in TB-related deaths, the proportions of patients who were successfully treated remained below the WHO target of 85% upon implementation of Xpert MTB/RIF. There was no improvement in treatment success rates even among patients with confirmed TB diagnosis. One explanation is the relatively high proportion (16%) of patients lost to follow-up which, contrary to our hypothesis, did not decrease with improved diagnosis, suggesting that a rapid and accurate test that confirms TB is not enough to improve patients’ (and health care workers’) commitment to treatment. Other possible explanations for not reaching the successful outcome target include false-positive clinical TB diagnosis in patients with other diseases, no or late detection of resistance to other drugs and a higher (although non-significantly) proportion of non-TB deaths among patients empirically treated for TB in the smear arm.

Both this study and a study from South Africa[6] showed that the benefits from Xpert as a diagnostic tool for TB detection are likely also undermined by frequent empirical treatment initiation.[12] This could also have happened in our study, contradicting our initial hypothesis, although even among patients with confirmed diagnosis no improvement in outcomes could be demonstrated.

The observed reduced case-fatality may have been a consequence of more advanced disease, on average, among patients diagnosed by smear microscopy in the baseline arm than among those diagnosed by Xpert in the intervention arm, especially among the subgroup of bacteriologically confirmed patients. Indeed, data from the pre-chemotherapy era show that there are considerably higher death rates among smear-positive than among smear-negative patients.[13]

Because of the stepped-wedge design, each month an increasing proportion of presumptive TB patients were tested with Xpert. Thus, in theory, the reduction in TB case-fatality could be the effect of other ongoing health-related improvements in the cities. We have no reason to believe that the case fatality reduction can be attributed to other interventions over such a short period. There were no overall, TB-related, and HIV/AIDS related health policy changes in either city. In a stepped-wedge study design, the arm per cluster is associated with time. In other words, both arm and study month are on the same causal pathway to the outcome. As our determinant of interest is arm, we could not simultaneously include calendar time as a covariate in our models. In the univariate model, calendar time was not associated with the occurrence of unsuccessful treatment outcomes (data not shown).

As we did observe a differential effect of HIV on bacteriologically confirmed TB case detection,[5] we also explored whether the effect of Xpert implementation on fatality rates differed by HIV status irrespective of whether the diagnosis had been bacteriologically confirmed. However, we did not observe such differential effect. Although smear-positive patients usually have more advanced TB disease, smear-negative HIV patients may have more advanced HIV disease, with lower CD4 counts that ultimately drive mortality.[14] Noteworthy, non-tested patients had risks of unsuccessful outcomes more similar to known HIV-positive patients than known HIV-negative patients, which calls for more inclusive testing of TB patients for HIV-status.

An additional patient and population benefit of the Xpert assay is the reduced time needed to detect resistance. Even though the National TB Program recommended maintaining patients with an Xpert test positive for rifampicin resistance on FLD treatment until results of culture-based susceptibility testing were available, referral to specialized resistance centres was reduced by a median of 2.5 months in the Xpert arm. For most of patients with confirmed resistance and started on second-line drug (SLD) treatment with a minimum duration of 18 months, treatment outcomes are not currently available. The referral delay could be further shortened if guidelines recommend immediate SLD treatment initiation upon a positive Xpert rifampicin resistance signal. This seems a plausible recommendation, given its high positive predictive value for resistance.[5,15] Reduced delay in drug-resistant TB diagnosis may have both patient and population benefits, since rapid initiation of effective treatment is likely to improve treatment outcomes and reduce transmission [3]

Our study had limitations. The “non-tested” diagnosis category may have introduced bias in the analysis. It corresponds to patients whose test could not be retrieved in the laboratory or reporting databases despite an extensive algorithm as previously reported.[5] An unknown proportion had not been tested, but a possible alternative explanation is incomplete linkage.[5] However, the characteristics of this group of patients are similar in the baseline and the intervention arm,[5] which argues against selection bias. As expected, the distributions of treatment outcomes among the “non-tested” patients were also similar in both arms.

As outcomes of “transferred-out” patients are unknown, they may introduce bias to the results. However, sensitivity analyses both excluding all transferred out and considering them as favourable outcomes did not change our conclusions. Cause of death could also have been a source of error in our analyses, but we minimized this error through reviewing death certificates whenever there was any discrepancy between the mortality and notification registries.

Finally, despite randomization of labs by the order in which Xpert was introduced, bias could have been introduced by the differential socioeconomic background of the population covered by these labs. However, in both cities, all neighbourhoods include patients from all socioeconomic strata. Nonetheless, we cannot guarantee that efforts engaged to reach defaulters are strictly similar in all clinics. Likewise, other potential confounders for poor outcomes could not be assessed, and although the stepped-wedge design and the short study period makes it unlikely that additional confounding explains our results it cannot be excluded.

Some questions remain unanswered. Our study did not allow the evaluation of the outcomes of patients who were not diagnosed with TB (but might have TB, i.e., false-negative diagnosis) in both arms. Xpert, which can reduce the number of false-negative diagnosis, might have a relevant effect in this group. In addition, statistically non-significant findings may be due to lack of power in small subgroups. The sample size for this study was calculated for showing increase in confirmation rates, not in outcomes.

Despite these limitations, to our best knowledge, this is the first study to report the effect of Xpert implementation on treatment outcomes in real-world programmatic conditions. In summary, we found that in the patients diagnosed during Xpert implementation, TB-related deaths were reduced by 35% and delay to referral to drug-resistant TB centres were reduced by 2.5 months, while no effect was observed on the proportion of patients with successful treatment outcomes, which may be due to relatively high loss to follow-up. We will keep monitoring effects of scaled up Xpert implementation in Brazil on case detection and treatment outcomes.

## Supporting Information

S1 CONSORT Checklist CONSORT Checklist. (PDF)

S1 Protocol Study protocol. (DOC)
