# Correction: Impact on Patients' Treatment Outcomes of XpertMTB/RIF Implementation for the Diagnosis of Tuberculosis: Follow-Up of a Stepped-Wedge Randomized Clinical Trial

**DOI:** 10.1371/journal.pone.0156471

**Published:** 2016-05-24

**Authors:** Anete Trajman, Betina Durovni, Valeria Saraceni, Alexandre Menezes, Marcelo Cordeiro-Santos, Frank Cobelens, Susan Van den Hof

There are a number of errors in the “aOR (95%CI)” columns for Tables 3 and 4. During secondary analysis, the authors observed 128 duplicate entries in the study database among 4054 included in the analyses. Despite observing the random distribution of duplicates, the authors performed all analyses again after removal of the 128 duplicates (leaving 3926 patients in the analyses) and found negligible effect on the outcome measures: aOR for arm associated with unfavourable outcome now 0.93 (0.80–1.09) compared to 0.93 (0.79–1.08) in the original article; while aOR for TB-attributed deaths is 0.69 (0.46–1.04) compared to 0.65 (0.44–0.97). Even though the 95% confidence interval of the aOR for TB attributed death now includes 1, the difference with the originally reported aOR and the width of its confidence interval are minimal, hence the conclusions from the results remain unchanged. Please see the correct Tables 3 and 4 here.

**Table 3:** Characteristics associated with unfavourable outcomes in a multilevel logistic regression model

Characteristics	Unfavourable† (n = 1181)	Successful (n = 2745)	crude OR (95%CI)	aOR (95%CI)
***Sex***				
Male	846 (33.2%)	1700 (66.8%)	1.5 (1.3–1.8)	1.5 (1.3–1.8)
Female	335 (24.3%)	1045 (75.7%)	1 (reference)	1 (reference)
***Age group (years)***				
0–14	18 (19.8%)	73 (80.2%)	0.76 (0.44–1.32)	0.69 (0.39–1.3)
15–29	704 (33.8%)	1378 (66.2%)	1.6 (1.3–2.0)	1.6 (1.2–2.0)
30–59	347 (26.9%)	943 (73.1%)	1.1 (0.90–1.5)	1.1 (0.86–1.4)
≥60	112 (24.2%)	351 (75.8%)	1 (reference)	1 (reference)
***HIV status***				
Negative	452 (21.2%)	1675 (78.8%)	1 (reference)	1 (reference)
Positive	171 (43.7%)	220 (56.3%)	2.9 (2.3–3.7)	3.1 (2.4–3.9)
Unknown	558 (39.6%)	850 (60.4%)	2.6 (2.2–3.0)	2.6 (2.2–3.0)
***City***				
Manaus	222 (28.5%)	556 (71.5%)	1 (reference)	1 (reference)
Rio	959 (30.5%)	2189 (69.5%)	1.1 (0.81–1.4)	1.3 (1.01–1.7)
***Type of diagnosis***				
Confirmed*	875 (30.9%)	1960 (69.1%)	1 (reference)	1 (reference)
Clinically diagnosed, negative test^§^	198 (28.5%)	496 (71.5%)	0.89 (0.73–1.1)	0.86 (0.71–1.04)
Clinically diagnosed, no test result	108 (27.2%)	289 (72.8%)	0.82 (0.65–1.04)	0.76 (0.59–0.97)
***Arm***				
Baseline	556 (31.3%)	1221 (68.7%)	1 (reference)	1 (reference)
Intervention	625 (29.1%)	1524 (70.9%)	0.92 (0.79–1.06)	0.93 (0.80–1.09)

*Positive smear in baseline arm and positive Xpert in intervention arm.

^§^Negative smear in baseline arm and negative Xpert in intervention arm.

†Unfavourable = loss to follow up, death from any cause, transfer out and resistance.

**Table 4:** Characteristics associated with TB-attributed death in a multilevel logistic regression model

Characteristics	TB-attributed death (n = 119)	Successful (n = 2745)	crude OR (95%CI)	aOR (95%CI)
***Sex***				
Male	83 (4.7%)	1700 (95.3%)	1.4 (0.95–2.1)	-
Female	36 (3.3%)	1045 (96.7%)	1.0 (reference)	
***Age group (years)***				
0–14	1 (1.3%)	73 (98.7%)	0.20 (0.03–1.6)	0.14 (0.02–1.01)
15–29	49 (3.4%)	1378 (96.6%)	0.54 (0.33–0.9)	0.44 (0.26–0.76)
30–59	46 (4.6%)	943 (95.4%)	0.74 (0.44–1.2)	0.57 (0.33–0.98)
≥60	23 (6.1%)	351 (93.9%)	1.0 (reference)	1.0 (reference)
***HIV status***				
Negative	25 (1.5%)	1675 (98.5%)	1.0 (reference)	1.0 (reference)
Positive	44 (16.7%)	220 (83.3%)	14.0 (8.1–24.2)	16.1 (9.2–28.3)
Unknown	50 (5.6%)	850 (94.4%)	4.1 (2.4–6.7)	4.3 (2.6–7.1)
***City***				
Manaus	21 (3.6%)	556 (96.4%)	1.2 (0.73–1.9)	1.96 (1.01–3.8)
Rio	98 (4.3%)	2189 (95.7%)	1.0 (reference)	1.0 (reference)
***Type of diagnosis***				
Confirmed*	81 (4.0%)	1960 (96.0%)	1.0 (reference)	
Clinically diagnosed, negative test^§^	25 (4.8%)	496 (95.2%)	1.2 (0.77–1.9)	-
Clinically diagnosed, no test result	13 (4.3%)	289 (95.7%)	1.1 (0.59–2.0)	
***Arm***				
Baseline	68 (5.3%)	1221 (94.7%)	1.0 (reference)	1.0 (reference)
Intervention	51 (3.2%)	1524 (96.8%)	0.60 (0.42–0.85)	0.69 (0.46–1.04)

OR = odds ratio, aOR = adjusted odds ratio

*Positive smear in baseline arm and positive Xpert in intervention arm

^§^ Negative smear in baseline arm and negative Xpert in intervention arm
